# Understanding Antipsychotic Polypharmacy in Bipolar Disorder: The Role of Long-Acting Injectable Antipsychotics in a Naturalistic Inpatient Setting

**DOI:** 10.1007/s11126-025-10250-7

**Published:** 2026-01-10

**Authors:** Nilgün Oktar Erdoğan, Bengü Yücens, Osman Mert Özcan, Fatmanur Ayhan, Selim Tümkaya

**Affiliations:** Department of Psychiatry, Pamukkale University, Denizli, Turkey

**Keywords:** Bipolar disorder, Long-acting injectable antipsychotics, Polypharmacy, Hospitalization

## Abstract

Antipsychotic (AP) polypharmacy is common in the treatment of bipolar disorder (BD), particularly during inpatient care. Long-acting injectable antipsychotics (LAIs) are frequently used to prevent relapse. However, their relationship with AP polypharmacy remains unclear. This study examined the prescription patterns in inpatients with BD, focusing on the relationship between clinical characteristics, LAI use, and AP polypharmacy. Sociodemographic and clinical data of 175 inpatients diagnosed with BD were retrospectively analyzed. Antipsychotic use at three time points (pre-hospitalization, at initial treatment, and at discharge) was recorded. Factors predicting discharge AP count, AP dosage, and the potential direct and indirect effects of pre-hospitalization LAI use were examined. Patients using LAIs before hospitalization had earlier onset of illness, more manic episodes, and higher AP burden compared to non-users. Discharge AP count and dosage were significantly associated with AP count at initial treatment, length of hospital stay, and manic episode frequency. LAI use demonstrated a trend-level indirect association with fewer APs at discharge, mediated by shorter hospital stays, and this pathway appeared stronger among patients with fewer prior manic episodes. AP polypharmacy is common in BD, especially among patients on LAI. However, the association is indirect and shaped by clinical factors. Regular review of treatment regimens may help minimize unnecessary polypharmacy.

**Supplementary Information:**

The online version contains supplementary material available at 10.1007/s11126-025-10250-7.

## Introduction

Bipolar disorder (BD) is a chronic mental disorder with mood episodes and a lifetime prevalence exceeding 1% globally [1], contributing to substantial morbidity, mortality, and functional impairment. Frequent relapses, cognitive deficits, repeated hospitalizations, and suicide risk necessitate long-term, adaptive treatment strategies [2]. First- and second-generation antipsychotics (APs) and mood stabilizers are first-line treatments in acute manic episodes. Over time, second-generation APs have come to the forefront not only in acute phases but also in maintenance treatments [3], and in some cases, have been replaced with mood stabilizers [4]. However, the role of AP agents in acute and long-term treatment has not been limited to monotherapy, and they are often included as members of combination therapies. Despite guideline recommendations to minimize polypharmacy, multiple drug regimens are frequently used in both acute and maintenance phases of BD.

Real-world data have shown a high prevalence of polypharmacy in BD [5]. The STEP-BD study revealed that 40% of patients used more than three psychotropic medications, and nearly one-fifth used more than four [6]. Antipsychotic polypharmacy, in particular, has been reported in approximately one-third of BD patients [7–9]. A study in inpatients observed that about half of the patients received AP polypharmacy [10], with usage increasing even further in acute units, mostly due to “as needed” intramuscular injections [11]. Concerns about the increased risk of side effects and mortality associated with polypharmacy compared to monotherapy [12] underscore the need for a careful evaluation of treatment strategies. Consistent with these concerns, major clinical guidelines provide limited and cautious recommendations for AP polypharmacy. The American Psychiatric Association Practice Guideline (2002) [13] and Canadian Network for Mood and Anxiety Treatments (2018) and the International Society for Bipolar Disorders [14] guidelines recommend combining an AP with a mood stabilizer, but not multiple APs, and low-dose AP combinations may be considered only for managing side effects. Meanwhile updated version of National Institute for Health and Care Excellence clinical guideline (2014) [15] advises against routine combined APs use except briefly during medication switching.

Long-acting injectable antipsychotics (LAIs) are recommended for high-risk patients due to their potential to reduce relapse rates and enhance adherence [16]. Evidence on their effectiveness, however, mixed. While some mirror-image studies have shown reductions in hospitalization rates following LAI initiation [17, 18], other real-world studies have not replicated these benefits [19]. A recent review found LAIs to be more effective in preventing relapses compared to placebo, but not superior to oral APs [20]. In contrast, a large-scale cohort study reported that the risk of rehospitalization (psychiatric and non-psychiatric) of LAI users was 30% lower than that of users of oral APs [21]. Taken together, although these findings suggest potential advantages of LAIs, the evidence base for their efficacy in BD remains more limited than in schizophrenia. Moreover, the use of LAI and AP polypharmacy have been widely studied in psychotic disorders; research specific to BD is relatively scarce.

The aim of this study was to examine the medication regimens and the burden of AP polypharmacy among inpatients with BD in a university hospital. Specifically, we focused on patients who had been using LAIs prior to admission and were hospitalized due to an acute episode. The study sought to explore how LAI treatment was managed during hospitalization and how it influenced AP polypharmacy at discharge. Through this investigation, we aimed to better understand the clinical challenges in managing this patient group and to test the hypothesis that LAI use is associated with increased AP polypharmacy during inpatient care.

## Method

Between January 1, 2022 and February 29, 2024, clinical data were retrospectively collected from patients admitted to inpatient psychiatry unit of a university hospital and were diagnosed with an acute episode of BD following psychiatric evaluation. The dataset included detailed sociodemographic and clinical characteristics such as the duration of disorder, total number of mood episodes, presence of psychotic features etc. Additionally, information regarding comorbid psychiatric and medical conditions, psychotropic medication use, and treatment adherence were recorded.

Hospitalization-related variables included the length of hospital stay (in days), and the number of psychotropic and AP medications prescribed at three time points: before hospitalization, during the initial treatment phase, and at discharge. To evaluate changes in pharmacological treatment over time, the number of prescribed psychotropics and APs was systematically compared across these three time points. Olanzapine equivalent doses of the APs used at discharge were calculated [22].

Inclusion criteria included being diagnosed with BD (type I or II) according to ICD-10 diagnostic criteria, hospitalization between 2022 and 2024, and availability of clinical and treatment records. Patients with a primary diagnosis of a psychotic disorder, those with an unclear diagnosis, or those primarily diagnosed with another psychiatric disorder, patients with a hospitalization shorter than seven days (as these admissions generally do not permit comprehensive assessment and treatment planning), patients admitted solely for maintenance ECT, and those who were voluntarily early discharged were excluded.

A total of 315 patients coded with the ICD-10 diagnosis of F31 (bipolar affective disorder) were hospitalized during the study period. After screening, although 81 patients had been initially coded with a BD diagnosis, examination of their discharge summaries indicated that their primary diagnoses were schizophrenia, schizoaffective disorder, other psychiatric disorders, or were unclear, and these patients were therefore excluded. Accordingly, 234 patients were identified as having a confirmed diagnosis of BD. Among these, 8 patients who had been admitted solely for maintenance electroconvulsive therapy (ECT), 6 patients with insufficient clinical data, and 36 patients whose hospitalization lasted fewer than seven days were excluded, resulting in 184 eligible patients. For individuals with multiple hospitalizations, only the most recent admission was included in the final analyses. Further exclusions were made among these 184 patients: 4 patients were experiencing their first episode and had used no psychotropic medication prior to admission, 4 had undocumented pre-hospitalization medication histories, and 1 patient was transferred to intensive care and thus had no documented discharge treatment plan. Two hospitalizations initially coded as hypomanic episodes were retained, as these admissions occurred either at patient request or for treatment adjustment. Ultimately, a total of 175 patients were included in the final analysis.

All statistical analyses were performed using IBM SPSS Statistics version 25.0. Continuous variables were given as mean ± standard deviations and categorical variables as numbers and percentages. The chi-square test was used to compare categorical variables. For independent group comparisons, an independent sample t-test was used when parametric test assumptions were met and Mann-Whitney U test was used when parametric test assumptions were not met. Pearson correlation analyses were conducted separately to examine the factors influencing the number of APs prescribed at initial treatment and at discharge. Variables that showed significant correlations with AP counts were included in multiple linear regression analyses to identify independent predictors.

Moderated mediation analysis was conducted using the Andrew F. Hayes PROCESS v4.3 macro, Model 7 [23]. It was structured to examine the possible effects of pre-hospitalization LAI use (X) on the number of APs prescribed at discharge (Y), with the number of APs at initial treatment (M1) and length of hospital stay (M2) as sequential mediators. The number of manic episodes (W) was included as a moderator based on its established role as an indicator of illness progression and severity in BD [24, 25] (which may influence prescribing patterns and treatment complexity). The number of pre-hospitalization APs was entered as a covariate to control for baseline treatment effect. In addition, the indication for the most recent hospitalization was also included as a covariate, and the results of these adjusted analyses are presented in the Supplementary Materials, which also include the correlation, regression, and moderated mediation analyses conducted using olanzapine-equivalent doses. For estimation of indirect and conditional effects, bias-corrected 95% confidence intervals were calculated with 5000 bootstrap samples. In all tests, the significance limit was accepted as *p* < 0.05, and indirect pathways were considered statistically significant when confidence intervals did not include zero. Ethics committee approval was obtained from Pamukkale University Clinical Research Ethics Committee on 30.04.2024 (E-60116787-020-521229).

## Results

### Sociodemographic and Clinical Characteristics

The sociodemographic and clinical characteristics of the sample are presented in Table 1. Of the patients, 126 (72.00%) were hospitalized due to manic episodes, 39 (22.28%) due to depressive episodes, 2 (1.14%) due to hypomanic episodes, and 8 (4.57%) due to mixed episodes. Regarding treatment adherence, 35.4% (*n* = 62) took their medications regularly, 35.4% (*n* = 62) missed their medications in the last 3 months, 21.1% (*n* = 37) had poor medication adherence for more than 3 months, 8.0% (*n* = 14) had no information. Among 39 patients using LAIs, 25.6% (*n* = 10) had regular medication adherence, 35.9% (*n* = 14) had irregular adherence in the last 3 months, 30.8% (*n* = 12) had no medication adherence in the last 3 months, and 7.7% (*n* = 2) had no information. Among the patients who used LAI and discontinued their treatment in the past, 47.36% discontinued treatment without medical advice, 13.15% discontinued the drug due to side effects, and the rest had no information. Comorbid psychiatric diagnoses were seen in 27.7% (*n* = 51), and the most common diagnoses were OCD (8.1%, *n* = 15), substance use disorder (5.4%, *n* = 10), anxiety disorder (6.2%, *n* = 11), and attention deficit hyperactivity disorder (4.8%, *n* = 9).

**Table 1:** Sociodemographic and clinical characteristics

Variable	Value
Sex, n (%)	
Female	102 (58.3%)
Male	73 (41.7%)
Bipolar type, %	
Type I	159 (90.9%)
Type II	16 (9.1%)
Age (years), Mean ± SD	40.16 ± 13.57
Marital status, n (%)	
Married	85 (48.6%)
Single	46 (26.3%)
Divorced/Widowed	17 (9.7%)
No information	27 (15.4)
Age at illness onset (years), Mean ± SD	27.61 ± 11.77
Duration of illness (years), Mean ± SD	13.24 ± 9.22
Length of stay, last hospitalization (days), Mean ± SD	26.54 ± 12.73
Psychotic features, n (%)	
Present	122 (69.7%)
Absent	36 (20.6%)
No information	17 (9.7%)
Number of prior hospitalizations, % 1/2/3/4/≥5	23.4%/22.3%/17.7%/12.0%/24.5%
Number of psychotropics at pre-hospitalization, Mean ± SD	2.74 ± 1.44
Number of antipsychotics at pre-hospitalization, Mean ± SD	1.36 ± 0.96
Number of psychotropics at initial treatment, Mean ± SD	3.09 ± 1.18
Number of antipsychotics at initial treatment, Mean ± SD	1.70 ± 0.85
Number of psychotropics at discharge, Mean ± SD	3.49 ± 1.25
Number of antipsychotics at discharge, Mean ± SD	2.06 ± 0.88

Notes: N: Number, %: Percentage, SD: Standard deviation, ECT: Electroconvulsive treatment

### Medication Use and Antipsychotic Polypharmacy

Medication use was analyzed at three time points: prior to hospitalization, during initial treatment, and at discharge (Table 1). The mean number of psychotropic medications increased progressively before hospitalization to discharge t(178) = −6.42, *p* < 0.001). Similarly, the mean number of APs rose (t(178) = −8.24, *p* < 0.001) over the same period.

Before hospitalization, 16.6% (*n* = 29) of patients were not receiving any AP, 45.1% (*n* = 79) were on one AP, 26.3% (*n* = 46) were on two APs, and 12.0% (*n* = 21) were on three or more APs. During initial treatment, including as-needed intramuscular injections, 47.4% (*n* = 83) of patients received one AP, 37.7% (*n* = 66) received two APs, and 10.3% (*n* = 18) received three APs. At discharge, 1.1% (*n* = 2) of patients were prescribed no APs, 23.4% (*n* = 41) were prescribed one AP, 49.7% (*n* = 87) were prescribed two APs, and 25.7% (*n* = 45) were prescribed three or more APs.

Both first- and second-generation LAIs were used in the sample. The most preferred pre-hospitalization LAI was paliperidone palmitate 1-month formulation (48.7%, *n* = 19). Other LAI options were aripiprazole extended-release injection (15.4%, *n* = 6), zuclopenthixol decanoate (12.8%, *n* = 5), paliperidone palmitate 3-monthly (10.3%, *n* = 4), risperidone long-acting injections (5.1%, *n* = 2), paliperidone 1 monthly and aripiprazole extended release injection (2.6%, *n* = 1), aripiprazole extended release injection and haloperidole decanoate combination (2.6%, *n* = 1) and another LAI for which no drug information was available (2.6%, *n* = 1). When it was examined how the treatment of patients with a history of LAI use before hospitalization was adjusted at discharge, it was observed that among the 39 patients with LAI history, 22 continued the same LAI, 8 switched to another LAI, and 9 discontinued LAI. The most common adjustment was the addition of an oral AP to existing LAI treatment (*n* = 16). The most common combined mood stabilizer with LAI is valproate (*n* = 21). Additionally, it was observed that patients were prescribed an average of 2.06 ± 1.67 different AP treatments before switching to LAI.

### Group Comparisons Based on LAI Use

A comparison was made between patients with (*n* = 39) and without (*n* = 136) a history of LAI use before hospitalization, and the results are presented in Table 2. As shown in Table 2, patients using LAI had a higher AP polypharmacy burden before admission, during initial treatment, and at discharge.

**Table 2:** Group comparisons of age and clinical characteristics between patients with and without LAI use before hospitalization

Variable	LAI users before hospitalization (*n* = 39) Mean ± SD	Others (*n* = 136) Mean ± SD	*P* value	Cohen’s d
Age	34.94 ± 10.58	41.66 ± 13.99	0.006*	−0.54
Age at onset of the disorder	22.00 ± 7.82	29.22 ± 12.22	0.001*	−0.70
Duration of illness	12.43 ± 6.62	13.47 ± 9.84	0.546	−0.12
Number of hospitalizations	5.10 ± 3.55	3.00 ± 2.46	0.001*	0.68
Number of mood episodes	6.64 ± 5.21	4.77 ± 3.72	0.042*	0.41
Number of manic episodes	3.41 ± 1.95	2.19 ± 1.58	0.001*	0.71
Length of hospital stay- last episode (days)	28.82 ± 12.59	25.88 ± 12.74	0.206	0.23
Number of psychotropics at pre-hospitalization	3.36 ± 1.38	2.56 ± 1.41	0.002*	0.57
Number of antipsychotics at pre-hospitalization	2.10 ± 1.01	1.16 ± 0.85	< 0.001*	1.03
Number of psychotropics at initial treatment	3.84 ± 1.24	2.88 ± 1.07	< 0.001*	0.86
Number of antipsychotics at initial treatment	2.38 ± 1.04	1.51 ± 0.68	< 0.001*	1.07
Number of psychotropics at discharge	3.82 ± 1.23	3.39 ± 1.25	0.064	0.35
Number of antipsychotics at discharge	2.41 ± 0.93	1.97 ± 0.84	0.006*	0.51
Mean dosage of antipsychotics at discharge, olanzapine equivalent dosages	36.92 ± 18.42	24.79 ± 15.43	< 0.001*	0.72

Notes: n = number, SD: Standard deviation, *: *p* < 0.05

### Predictors of Antipsychotic Polypharmacy

The number of APs prescribed at discharge was significantly correlated with a range of clinical and medication-related variables. Among clinical factors, it was positively correlated with the number of manic episodes (*r* = 0.312, *p* < 0.001), the length of hospital stay (*r* = 0.297, *p* < 0.001), and having a history of psychotic episodes (*r* = 0.158, *p* = 0.046). A significant negative correlation was found with age at onset of psychiatric illness (*r* = − 0.222, *p* = 0.003). In terms of medication-related variables, the number of APs at discharge was significantly correlated with the number of APs prescribed at initial treatment (*r* = 0.467, *p* < 0.001) and the total number of psychotropic medications at initial treatment (*r* = 0.341, *p* < 0.001). It was also positively associated with the number of APs before hospitalization (*r* = 0.186, *p* = 0.014) and with LAI use before hospitalization (*r* = 0.208, *p* = 0.006).

The number of APs prescribed at initial treatment was significantly correlated with several clinical factors. It was negatively associated with age at onset of psychiatric illness (*r* = − 0.164, *p* = 0.031) and positively associated with the number of manic episodes (*r* = 0.261, *p* = 0.001), number of psychiatric hospitalizations (*r* = 0.263, *p* < 0.001). Also, significant positive correlations were observed with history of LAI use before hospitalization (*r* = 0.423, *p* < 0.001), number of APs before hospitalization (*r* = 0.372, *p* < 0.001), and number of psychotropic medications before hospitalization (*r* = 0.257, *p* = 0.001). Correlation and regression analyses conducted using olanzapine-equivalent doses are presented in the Supplementary Materials, and the regression results are summarized in STable 1.

Multivariate linear regression analyses were conducted using the variables identified in the prior correlation analyses as independent predictors. The number of APs at discharge and the number of APs in the initial treatment were used as dependent variables. As presented in Table 3, the results showed that the number of APs at discharge was significantly predicted by the number of APs in the initial treatment, the number of manic episodes, the length of hospital stay. Additionally, the number of APs in the initial treatment was significantly associated with LAI use before hospitalization and the number of APs prescribed before hospitalization. All Variance Inflation Factor (VIF) values were below the commonly accepted threshold of 5.

**Table 3:** Regression results predicting APs at initial treatment and APs prescribed at discharge

Dependent variable	Independent variables		Unstandardized Coefficients		Standardized Coefficients	t	VIF	Sig.	Adjusted R2
			B	Std. Error	Beta				
Number of APs at discharge	Number of APs at initial treatment, Number of manic episodes, Length of hospital stay	Number of APs at initial treatment	0.401	0.071	0.391	5.625	1.082	< 0.001	0.291
		Length of hospital stay	0.016	0.005	0.236	3.477	1.029	0.001	
		Number of manic episodes	0.082	0.035	0.165	2.363	1.087	0.019	
Number of APs at initial treatment	LAI use before hospitalization, Number of APs before hospitalization	LAI use before hospitalization	0.706	0.161	0.333	4.389	1.185	< 0.001	0.225
		Number of APs before hospitalization	0.214	0.067	0.244	3.208	1.185	0.002	

Notes: LAI: Long-acting injectable antipsychotics, AP: Antipsychotic

### Mediation and Moderation Results

The results of the mediation model regression analyses are presented in Table 4. In the first regression analysis, LAI use before hospitalization significantly predicted the number of APs at initial treatment. While the number of manic episodes was not a significant predictor, the covariate, the number of APs used before hospitalization, was significantly associated with the outcome. The interaction between LAI use and manic episodes was not statistically significant.

**Table 4:** Moderated mediation model regression results

Dependent Variable	Independent Variables	B	Std. Error (SE)	t	Sig.	95% CI (LL, UL)
M1	Constant	1.15	0.13	8.60	< 0.001*	0.88, 1.41
	X	0.57	0.27	2.11	0.036*	0.04, 1.11
	W	0.06	0.04	1.46	0.145	−0.02, 0.14
	X × W Interaction	0.01	0.08	0.16	0.875	−0.14, 0.16
	Covariate	0.20	0.07	3.10	0.002*	0.07, 0.33
	*R* = 0.493, R² = 0.243, F(4, 170) = 13.70, *p* < 0.001 ΔR² for interaction (X×W) = 0.0001, *p* = 0.875					
M2	Constant	26.26	2.19	12.01	< 0.001*	21.95, 30.58
	X	−10.14	4.43	−2.29	0.023*	−18.90, −1.39
	W	−0.26	0.67	−0.38	0.704	−1.58, 1.07
	X × W Interaction	3.88	1.23	3.14	0.002*	1.44, 6.32
	Covariate	0.16	1.07	0.15	0.881	−1.95, 2.27
	*R* = 0.280, R² = 0 0.078, F(4, 170) = 3.63, *p* = 0.007 ΔR² for interaction (X×W) = 0.053, *p* = 0.002					
Y	Constant	0.85	0.17	4.88	< 0.001*	0.51,1.20
	X	−0.003	0.16	−0.02	0.986	−0.32, 0.31
	M1	0.45	0.08	5.86	< 0.001*	0.30, 0.60
	M2	0.02	0.00	3.68	< 0.001*	0.01, 0.03
	Covariate	0.00	0.07	0.06	0.956	−0.13, 0.14
	*R* = 0.525, R² = 0.276, F(4, 170) = 16.23, *p* < 0 0.001					

Notes: X: LAI use before hospitalization, W: number of manic episodes, M1: number of APs at initial treatment, M2: length of hospital stay, Y: number of APs at discharge. Covariate (number of APs before hospitalization) was controlled in all equations. ΔR²: R² change, B: unstandardized regression coefficient; SE: standard error; LLCI: lower bound of 95% confidence interval; ULCI: upper bound of 95% confidence interval, *: *p* < 0.05

In the second model, the main effect of LAI use indicates shorter hospital stays and this association varied by the number of manic episodes, confirming a moderation effect. Neither the number of manic episodes alone nor the covariate significantly predicted the duration of hospital stay. At low levels of the number of manic episodes, LAI use had a negative effect on the duration of hospital stay and showed a trend toward statistical significance (B = − 6.26, *p* = 0.074). At high levels, LAI use had a positive effect with a trend toward statistical significance (B = 5.38, *p* = 0.058), whereas at moderate levels it was nonsignificant (B = − 2.38, *p* = 0.393).

In the final regression model, LAI use before hospitalization did not have a significant direct effect on the number of APs at discharge. However, both mediators, APs at initial treatment and length of hospital stay, were significant positive predictors of AP count at discharge. The covariate did not contribute significantly in this model.

The indirect effect of LAI use on the number of APs at discharge through the number of APs at initial treatment was statistically significant at all levels of the number of manic episodes including low (Effect = 0.26, 95% CI [0.03, 0.53]), moderate (Effect = 0.27, 95% CI [0.08, 0.50]), and high (Effect = 0.28, 95% CI [0.09, 0.55]). However, the index of moderated mediation for this pathway was not statistically significant (Index = 0.005, 95% CI [–0.07, 0.11]), indicating that the strength of this indirect effect did not significantly vary across levels of manic episodes (No moderation by number of manic episodes).

The indirect effect of LAI use before hospitalization through length of hospital stay was significant only at low levels of the number of manic episodes (Effect = − 0.10, 95% CI [–0.23, − 0.01]). This suggests that, among patients with fewer manic episodes, pre-hospitalization LAI use was associated with shorter hospital stays, which in turn predicted fewer APs at discharge. However, at high levels of the number of manic episodes, LAI use is associated with longer hospital stay and more APs at discharge, and this effect is statistically significant (Effect = 0.09, 95% CI [0.02, 0.21]). This indirect effect was not significant at moderate levels (Effect = − 0.04, 95% CI [–0.12, 0.03]). Moreover, the index of moderated mediation for this pathway was statistically significant (Index = 0.06, 95% CI [0.02, 0.13]), indicating that the strength of the indirect effect via length of hospitalization varied significantly by manic episode count, supporting a moderated mediation effect.

**Fig. 1:**
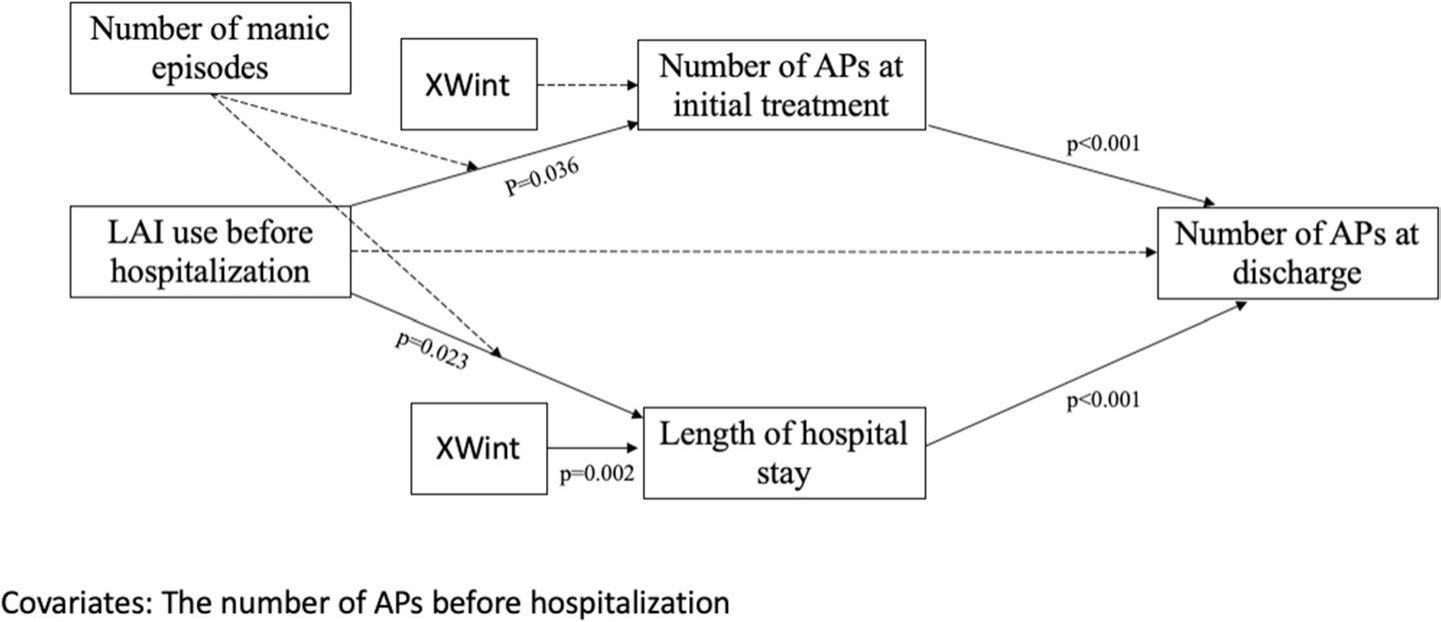
Moderated mediation model (PROCESS Model 7). Notes: LAI use before hospitalization (X) affects the number of APs at discharge (Y) through the number of APs at initial treatment (M1) and length of hospital stay (M2), moderated by the number of manic episodes (W), with with number of APs before hospitalization as a covariate. Solid lines represent statistically significant pathways (*p* < 0.05), while dotted lines represent non-significant paths. The interaction terms (XWint) indicate moderation effects

In summary, among patients with fewer manic episodes, LAI use showed a trend-level indirect reduction in APs at discharge via shorter hospital stays. However, among those with more manic episodes, the trend-level indirect effect reversed, with LAI use linked to more APs at discharge via longer hospital stays. This conditional moderated mediation model is illustrated in Fig. 1. When the analyses were repeated using olanzapine-equivalent doses instead of the number of APs prescribed at discharge, the findings remained consistent and supported the main results, which are presented in the Supplementary Materials. Additionally, when admission indications were grouped into manic/hypomanic/mixed versus depressive episodes and added as an additional covariate, this variable affected only the final model predicting the total AP load at discharge. Adding these covariates did not change the main pattern or significance of the moderated mediation results. Supplementary Tables 2a and 2b summarize these findings.

## Discussion

This study examined the clinical correlates and predictors of AP polypharmacy at discharge among inpatients with BD, with a particular focus on LAI use before hospitalization. Our findings showed that LAI users presented with a more severe clinical profile, characterized by earlier illness onset, a higher number of manic episodes, and greater AP burden throughout hospitalization. The number of APs prescribed at discharge was significantly associated with AP load during initial treatment, length of hospitalization, and the number of manic episodes. Importantly, LAI use demonstrated a trend-level indirect association with fewer APs at discharge, mediated by shorter hospital stays, and this pathway appeared stronger among patients with fewer prior manic episodes. Specifically, LAI use was associated with shorter hospital stays in patients with fewer manic episodes, but longer stays among those with more manic episodes. These results suggest that the impact of LAI use on discharge prescribing patterns is shaped by both disease burden and treatment experiences across the course of hospitalization.

In the present sample, more than half of the patients were discharged with AP polypharmacy. This pattern aligns with real-world studies showing high polypharmacy rates in BD and other psychotic disorders [26, 27]. Although clinical guidelines generally discourage AP polypharmacy, AP combinations remain especially common in acute inpatient settings. Also, patients hospitalized with manic episodes are more likely to receive AP combinations compared to outpatients [9, 27]. Previous studies have shown that earlier age at onset [28], longer illness duration [29], and greater symptom severity [30], involuntary treatment [31], more frequent hospital admissions [32], and longer duration of previous admissions [33] are associated with polypharmacy. Additionally, higher AP dosage [34], LAI use [35] are also linked to increased polypharmacy risk [36]. Consistent with the literature, our study also found that AP polypharmacy was associated with the number of manic episodes, length of hospital stay, having history of psychotic episode and earlier onset of disorder.

We found that the frequency of polypharmacy increased throughout hospitalization and the strongest predictor of AP polypharmacy at discharge was the number of APs prescribed at initial treatment suggesting a clinical tendency toward more complex pharmacological regimens during acute inpatient care. This raises a critical concern of whether clinicians tend to add APs rather than switch or discontinue them, particularly in patients who are already on polypharmacy. Clinicians’ tendency to add APs rather than combine mood stabilizers or simplify existing regimens may have several explanations. The difference observed between inpatients and outpatients suggests that episode or disorder severity is an important factor in polypharmacy. In acute inpatient settings, clinicians often need to stabilize patients rapidly and use APs, which allow faster titration, more immediate control of agitation, psychosis, and severe mood symptoms. In contrast, mood stabilizers require serum level monitoring, slower titration, and have narrower therapeutic windows. Moreover, an additional factor may be that mood stabilizers, many of which are older and generic medications, receive minimal marketing compared to newer APs. These factors may explain the gap between guideline-based recommendations and clinical practice, highlighting the complexity of real-world decision-making in acute psychiatry settings.

Beyond patients’ individual clinical characteristics, broader contextual and organizational factors also may affect prescribing patterns. System-level factors such as poor continuity of care [37, 38] and prescriber-level factors such as demographics, clinical workload, and prescribing habits may further contribute to polypharmacy [39–41], as a pragmatic strategy in the context of anticipated poor adherence and limited post-discharge follow-up. While such concerns are understandable in the context of acute psychiatric care, they may inadvertently reinforce a bias against medication simplification. Notably, studies examining deprescribing interventions have shown that reducing polypharmacy does not worsen clinical outcomes [42, 43]. The real-world data show that patients with BD frequently receive multiple psychotropic medications [26, 27, 44, 45]. Furthermore, the clinical benefit of AP polypharmacy appears limited, as it may increase relapse risk, side-effect burden, and poorer overall outcomes without significant improvements in efficacy [12].

In the present study, a similar tendency toward maintaining AP combinations may be observed in Türkiye, where short inpatient stays, limited outpatient follow-up capacity, and high patient load may further encourage rapid stabilization over gradual medication optimization. These systemic and cultural factors provide important context for understanding the persistence of polypharmacy practices observed in our study. To our knowledge, this tendency to maintain existing AP combinations has not been previously reported in BD populations. Recognizing this prescribing pattern is essential and may prompt clinicians to reconsider unnecessary AP use and help bridge the gap between guideline recommendations and real-world practice.

Another important finding is that LAIs were rarely discontinued during hospitalization, even in cases where further treatment adjustments were required. Rather than optimizing existing LAI regimen, clinicians often added oral APs with a different active substance to manage acute symptoms, to support treatment until the LAI’s therapeutic effect was established, or in cases of partial response to LAI treatment. In addition, a strong reliance on LAIs for maintaining adherence and preventing relapse may further reinforce this practice. As a result, the number of APs prescribed tends to increase from admission to discharge, which may cause the negative consequences mentioned above. Therefore, treatment planning should aim to balance short-term stabilization with regimen simplicity, tolerability, and sustained patient engagement after discharge [46].

Our findings showed that patients receiving LAI before hospitalization had an earlier age at illness onset and a higher number of manic episodes, and more likley to receive AP combinations during hospitalization. This pattern likely reflecting clinician selection bias toward prescribing LAIs for patients with more severe or recurrent disorders. Similar trends have been reported in previous studies including a multi-center study conducted in Asia, which reported that, although LAI use among individuals with BD remains relatively low, those who receive LAI had a higher overall medication burden [47].

While earlier expert opinions recommended the use of LAI in patients with multiple episodes, poor treatment adherence and frequent attacks [16], current approaches support initiating LAIs earlier in the disease course, ideally from the first episode [48]. Our findings are consistent with these up-to-date recommendations and provide empirical support for the potential benefits of earlier LAI use. Our moderated mediation model showed that among patients with fewer manic episodes, LAI use was associated with shorter length of hospital stay and lower AP burden at discharge, suggesting that early initiation may have potential to simplify treatment early in the illness course. In contrast, in patients with many manic episodes, LAI use was associated with longer stays and higher discharge polypharmacy, likely reflecting more refractory illness requiring intensified treatment. These findings highlight the need to tailor LAI strategies to illness chronicity, aiming to prevent unnecessary medication burden in chronic cases.

In our sample, poor adherence was common regardless of LAI use, underscoring that LAIs alone cannot fully resolve the adherence problem even if they are often used to improve adherence [49, 50]. Polypharmacy itself is a major contributor to nonadherence by increasing regimen complexity and side-effect burden. As the number of medications increases, treatment adherence deteriorates, and the chances of achieving and maintaining remission decline [51]. Side effects, perceived efficacy, and patient preferences also influence treatment compliance, underscoring the value of shared decision-making into clinical care [52]. In addition, systemic factors such as insufficient outpatient follow-up, limited access to community mental health services, and delays in hospital admission may also influence treatment plans. These problems need to be solved in cooperation with health authorities.

This study has some important limitations. First, since the data were collected from retrospective hospital records, longitudinal follow-up was not possible. Given the retrospective observational design, causal inferences can not be drawn; the findings reflect associations rather than causation. Second, the study was conducted in a single tertiary care center and included only inpatients, and the relatively small sample size reduces the generalizability of the findings to other settings or patient groups. Also, including only the most recent hospitalization during the study period may have led to an underrepresentation of individuals with rapid-cycling BD, potentially limiting the generalizability of the findings. Short-term hospitalizations were excluded to ensure more stable data on treatment processes. While this criterion increases internal consistency, it likely resulted in the selection of clinically more severe and treatment-resistant sub-groups, as reflected by the high average number and doses of APs. Third, the lack of standardized clinical assessments, such as symptom severity rating scales or functional outcome assessment scales, prevented precise assessment of patients’ clinical status at baseline and at discharge. Although we attempt to infer disorder burden from previous clinical course variables (e.g., total number of episodes, total number of manic episodes, early age of illness onset, previous AP exposure), these indicators are not a substitute for validated clinical scales. Another characteristic limiting the generalizability of this single-center study is that clinicians’ familiarity with the use of LAIs, which may have influenced both prescription practices, treatment outcomes and results. Finally, although moderated mediation analyses were conducted to explore potential indirect pathways between clinical variables and discharge outcomes, these analyses are limited by sample size and the inherent constraints of non-randomized designs. Larger-scale, prospective studies are needed to validate these findings and elucidate the dynamic interactions between patient characteristics, treatment decision-making processes, and long-term clinical outcomes. Understanding these dimensions will be important for optimizing AP treatment strategies and minimizing unnecessary medication burden in individuals with BD.

## Conclusion

In conclusion, this study highlights the high prevalence of AP polypharmacy in BD and its association with initial treatment intensity. While LAI use affects discharge prescribing patterns indirectly through early AP load and length of hospital stay, this relationship is moderated by the number of manic episodes. A key methodological strength of the study is the use of a moderated mediation model, which allowed for a nuanced examination of indirect and conditional effects in treatment complexity. This analytic approach provides insights beyond those of descriptive studies and underscores the multifactorial nature of prescribing practices in real-world inpatient psychiatric care. In clinical practice, to avoid the risks of polypharmacy, including poor adherence and increased side effect burden. Clinicians should periodically reassess LAI-based polypharmacy, especially after stabilization, to ensure optimal therapeutic outcomes.

## Supplementary Information

Below is the link to the electronic supplementary material.

Supplementary Material 1
