# Supplementary material for: Understanding Antipsychotic Polypharmacy in Bipolar Disorder: The Role of Long-Acting Injectable Antipsychotics in a Naturalistic Inpatient Setting
Source: Psychiatr Q. 2026 Jan 10;97(2):503–18. doi: 10.1007/s11126-025-10250-7 (PMC13328225; doi:10.1007/s11126-025-10250-7)
Supplement: Supplementary file 1 — Supplementary Material 1 [file 11126_2025_10250_MOESM1_ESM.docx]

**Declaration of Competing Interest**

The authors have no conflicts of interest to declare that are relevant to the content of this article.
